# Infective pneumonia following the use of tumor necrosis factor-α inhibitors in inflammatory bowel disease patients: A real-world disproportionality analysis of the FDA Adverse Event Reporting System (FAERS) database

**DOI:** 10.1371/journal.pone.0317242

**Published:** 2025-08-05

**Authors:** Qinhui Tang, Xiaowei Tang, Wenmeng Yin, Yantong Li, Xiaolin Zhong

**Affiliations:** 1 Department of Gastroenterology, the Affiliated Hospital of Southwest Medical University, Luzhou, China; 2 Nuclear Medicine and Molecular Imaging Key Laboratory of Sichuan Province, Luzhou, China; Pennsylvania State University Hershey Medical Center, UNITED STATES OF AMERICA

## Abstract

**Background:**

Patients with inflammatory bowel disease may develop infective pneumonia after using tumor necrosis factor-α inhibitors(TNFis). Due to the limitations of clinical trials, the occurrence of infective pneumonia in patients with inflammatory bowel disease using tumor necrosis factor-α(TNF-α) inhibitors remains uncertain. This article primarily explores the relationship between TNF-α inhibitors and adverse events(AEs) related to infective pneumonia in the US Food and Drug Administration (FDA) Adverse Event Reporting System (FAERS) database.

**Methods:**

We collected data from the FAERS database, extracting reports for each TNF-α inhibitor from their market launch until the first quarter of 2024 (infliximab, adalimumab, certolizumab pegol, and golimumab) and assessing infective pneumonia associated with TNF-α inhibitors using disproportionality analysis.

**Results:**

After removing duplicate reports, a total of 7176 reports were included. Infliximab and adalimumab exhibited the highest incidence of infective pneumonia-related adverse events, occurring in 3,858 and 2,819 cases, respectively, whereas certolizumab pegol and golimumab showed lower incidences with only 297 and 202 cases. Infliximab had the most positive signals, totaling 10, including tuberculosis, pulmonary tuberculosis, pneumocystis jirovecii pneumonia, histoplasmosis, pneumonia bacterial, pneumonia legionella, bronchopulmonary aspergillosis, tuberculous pleurisy, pneumonia cryptococcal, blastomycosis. Golimumab had seven positive signals, including pneumonia, tuberculosis, pulmonary tuberculosis, bronchopulmonary aspergillosis, pneumonia legionella, pneumonia bacterial, and COVID-19 pneumonia. Certolizumab pegol had only two positive signals: pneumonia and pneumonia klebsiella. However, adalimumab did not show signals of infective pneumonia.

**Conclusion:**

Except for adalimumab, the other three TNF-α inhibitors showed positive signals related to infective pneumonia, with tuberculosis-related diseases being the most common. Our study provides important insights for healthcare professionals, which can help reduce the occurrence of infective pneumonia associated with TNF-α inhibitors.

## 1. Introduction

Inflammatory bowel disease (IBD) is a chronic inflammatory disorder primarily affecting the digestive system, with Crohn’s disease (CD) and ulcerative colitis (UC) as its main types, and is caused by the interaction of environmental, genetic, infectious, immune, and other factors [1,2]. The pro-inflammatory cytokine tumor necrosis factor-α(TNF-α) is the mediator of inflammatory bowel disease. TNF-α plays a central role in the pathogenesis of IBD by promoting the recruitment of immune cells, enhancing epithelial apoptosis, and sustaining the activation of NF-κB signaling pathways, thereby perpetuating chronic intestinal inflammation [3]. The management of IBD often necessitates the use of immunosuppressive therapies to mitigate inflammation and maintain disease control. Among these therapies, TNF-α inhibitors have emerged as pivotal agents, revolutionizing the treatment landscape by targeting key cytokines involved in the inflammatory cascade [4]. Based on safety and efficacy data from clinical trials, the FDA has approved four drugs for IBD, including infliximab, adalimumab, certolizumab pegol, and golimumab [5].

Despite their efficacy, TNF-α inhibitors pose notable safety concerns, particularly infectious complications due to their immunomodulatory effects [6,7]. Due to the immunosuppressive effects of TNF-α inhibitors, individuals who use these medications face a higher risk of infections and may experience a more severe progression of their condition compared to healthy individuals who are not on immunosuppressive treatments. Infective pneumonia represents a significant clinical challenge in IBD patients receiving TNF-α inhibitors, stemming from both disease-related immunosuppression and drug-induced alterations in immune response [8]. Especially in countries and regions with a high prevalence of mycobacterium tuberculosis, the infection risk with TNF-α inhibitors is further increased [9]. The incidence of IBD is gradually increasing worldwide, even in regions with traditionally lower rates [10]. Consequently, the demand for TNF-α inhibitors is also expected to rise, so we should pay particular attention to TNF-α inhibitor-related infective pneumonia.

While clinical trials have provided initial safety data on TNF-α inhibitors, real-world evidence from pharmacovigilance studies offers critical insights into the occurrence, characteristics, and management of infective pneumonia. This paper aims to conduct a comprehensive pharmacovigilance analysis focusing on infective pneumonia following TNF-α inhibitor therapy in IBD patients. Currently, the FDA Adverse Event Reporting System is the largest global repository for spontaneously reported adverse events. These data are utilized to analyze adverse events, identify safety signals, and reassess the post-market safety profiles of medications [11].

## 2. Methods

### 2.1. Data source

**Fig 1:**
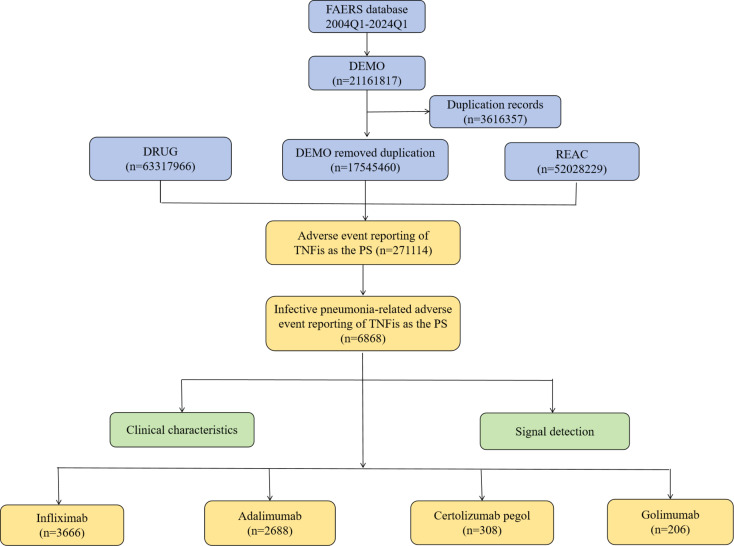
The flowchart of data extraction from the FAERS database.

FAERS is a database maintained by the US Food and Drug Administration, designed to collect and store adverse event reports related to drugs and pharmaceutical products. The FAERS comprises seven datasets, including patient demographic and administrative information (DEMO), drug information (DRUG), adverse events (REAC), patient outcomes (OUTC), report sources (RPSR), therapy start states and end dates for reported drugs (THER), and indications for drug administration (INDI) [12]. The research workflow is illustrated in Fig 1. We initially obtained 21,161,817 case records. After removing duplicate entries, we screened and identified 17,545,460 unique case reports. Focusing on TNF-α inhibitors as the primary suspected (PS) drugs, we identified 271,114 patients reporting drug-related adverse events, including 6,868 cases of infective pneumonia. Ethical committee approval is not required since this is a database-based observational study that does not involve drug treatments or diagnostic tests.

### 2.2. Data collection

We extracted reports for each TNF-α inhibitor from the market launch to the first quarter 2024. The drug codes reported in the events include the primary suspected drug (PS), secondary suspected drug (SS), concomitant drug (C), and interacting drug (I) [13]. We selected the role _cod as PS. The generic and brand names were used to identify adverse events related to TNF-α inhibitors. Due to the spontaneous nature of the reports, duplication is unavoidable; we performed duplicate data removal according to FDA recommendations [14]. In the FAERS database, adverse events are labeled with preferred terms (PTs) according to the Medical Dictionary for Regulatory Activities (MedDRA); these terms are different descriptors of a single medical concept. An initial broad screening for “Infective pneumonia” was conducted using standardized MedDRA analysis queries (SMQ) [Code: 20000231], resulting in the preliminary identification of 83 PTs (S1 Table).

### 2.3 Disproportionality analysis

Our analysis applied complementary signal detection methodologies, the reporting odds ratio (ROR) and the Bayesian confidence propagation neural network (BCPNN) to identify TNF-α inhibitor-associated AEs. Based on established pharmacovigilance criteria, a positive signal was confirmed when the lower 95% confidence limit of the reporting odds ratio (ROR) exceeded 1 with at least three reported adverse events. Similarly, the Bayesian Confidence Propagation Neural Network (BCPNN) method defined a significant signal when the Information Component’s 95% lower confidence bound surpassed zero with three or more documented cases (S2 Table). A statistically significant signal was established when both predefined criteria were satisfied. This study primarily used R software and Microsoft Excel for statistical analysis.

## 3. Results

### 3.1. Descriptive analysis

From the launch date of each TNF-α inhibitor until the first quarter of 2024, 7176 reports were included after removing duplicates. In this study, we excluded Preferred Terms with fewer than three occurrences. Infliximab and adalimumab exhibited the highest incidence of infective pneumonia-related adverse events, occurring in 3,858 and 2,819 cases, respectively, whereas certolizumab pegol and golimumab showed lower incidences with only 297 and 202 cases. (Table 1)

**Table 1:** Total reports of TNF-α inhibitors associated with IBD indication.

Primary suspected drug	Period	AEs of target drug	Infective pneumonia-related AEs
Infliximab	2004Q1-2024Q1	425756	3858
Adalimumab	2004Q1-2024Q1	565993	2819
Certolizumab pegol	2008Q2-2024Q1	40015	297
Golimumab	2009Q2-2024Q1	11345	202
Total			7176

### 3.2. Population characteristics

The characteristics of IBD patients with infective pneumonia-related AEs for different TNF-α inhibitors are presented in Table 2. In IBD patients taking TNF-α inhibitors, infective pneumonia-related AEs typically occurred in the age groups of 18–64.9 years (47.6%). Males were more likely to experience infective pneumonia-related AEs in patients taking infliximab and golimumab. In contrast, females were more likely to experience adalimumab and certolizumab pegol in patients taking them. The United States and Canada were the leading countries in reporting infective pneumonia-related AEs, accounting for 29.6% and 28.1%, respectively. Reports were primarily submitted by consumers (n = 2670, 38.9%), physicians (n = 1897, 27.6%), and health professionals (n = 1079, 15.7%). The serious outcomes of adverse events associated with TNF-α inhibitors were primarily hospitalization(n = 2793, 40.7%). Overall, the incidence of death, life-threatening, and disability outcomes associated with TNF-α inhibitors was relatively low, accounting for 5.5%, 2.0%, and 0.2%, respectively.

**Table 2:** Patient characteristics and incidence (number and percentage) of infective pneumonia adverse events associated with TNF-α inhibitors.

	Overall (n = 6868)	Infliximab n=(3666)	Adalimumab n=(2688)	Certolizumab pegol n=(308)	Golimumab n=(206)
**Age**					
<18	308(4.5%)	257 (7.0%)	35 (1.3%)	4 (1.3%)	12 (5.8%)
18 ~ 64.9	3272(47.6%)	1693 (46.2%)	1278 (47.5%)	187 (60.7%)	114 (55.3%)
65 ~ 85	995(14.5%)	389 (10.6%)	523 (19.5%)	47 (15.3%)	36 (17.5%)
>85	48(0.7%)	17 (0.5%)	23 (0.9%)	2 (0.6%)	6 (2.9%)
Unknown	2245(32.7%)	1310 (35.7%)	829 (30.8%)	68 (22.1%)	38 (18.4%)
**Gender**					
Male	2893(42.1%)	1557 (42.5%)	1122 (41.7%)	111 (36.0%)	103 (50.0%)
Female	3164(46.1%)	1383 (37.7%)	1503 (55.9%)	191 (62.0%)	87 (42.2%)
Others	811(11.8%)	726 (19.8%)	63 (2.3%)	6 (1.9%)	16 (7.8%)
**Reporter country**					
United States	2031(29.6%)	609(16.6%)	1133(42.2%)	274(89.0%)	15(7.3%)
Canada	1932(28.1%)	1644 (44.8%)	185(6.9%)	12(3.9%)	91(44.2%)
United Kingdom	35(0.5%)	28 (0.8%)	7 (0.3%)	0 (0%)	0 (0%)
Others	2870(41.8%)	1385(37.8%)	1363(50.7%)	22(7.1%)	100(48.5%)
**Reporter occupation**					
Pharmacist	114(1.7%)	54 (1.5%)	40 (1.5%)	11 (3.6%)	9 (4.4%)
Physician	1897(27.6%)	1137 (31.0%)	608 (22.6%)	70 (22.7%)	82 (39.8%)
Health professional	1079(15.7%)	915 (25.0%)	98 (3.6%)	18 (5.8%)	48 (23.3%)
Consumer	2670(38.9%)	781 (21.3%)	1728 (64.3%)	117 (38.0%)	44 (21.4%)
Others	1108(16.1%)	779(21.2%)	214(8.0%)	92(29.9%)	23 (11.2%)
**Outcomes**					
Hospitalization	2793(40.7%)	1176 (32.1%)	1421 (52.9%)	98 (31.8%)	98 (47.6%)
Disability	11(0.2%)	2 (0.1%)	8 (0.3%)	0 (0%)	1 (0.5%)
Life-Threatening	139(2.0%)	70 (1.9%)	60 (2.2%)	0 (0%)	9 (4.4%)
Death	381(5.5%)	174 (4.7%)	186 (6.9%)	11 (3.6%)	10 (4.9%)
Other outcomes	3544(51.7%)	2244(61.2%)	1013(37.7%)	199(64.6%)	88 (42.7%)

### 3.3. Disproportionality analysis

**Fig 2:**
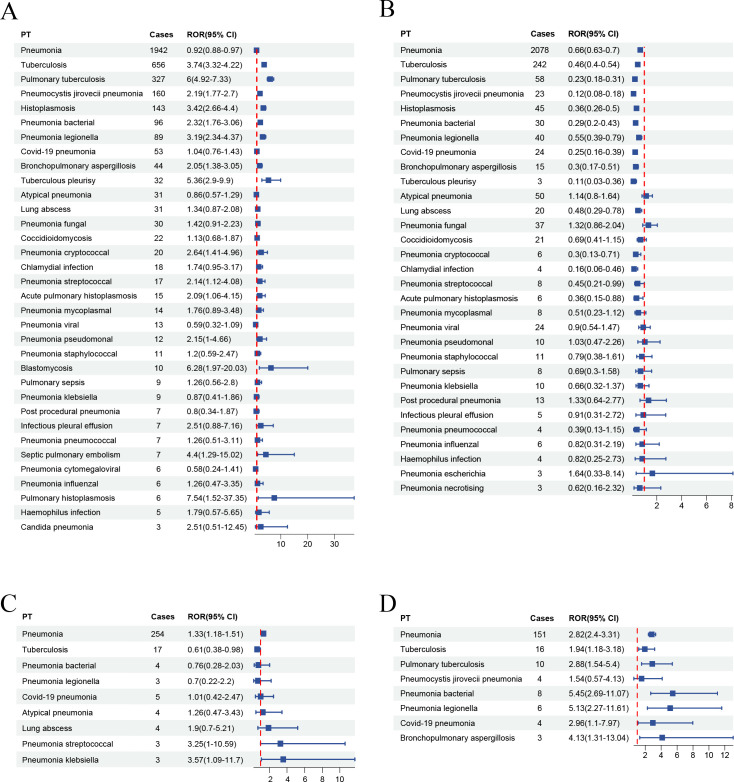
The forest plot of Preferred Terms for four TNF-α inhibitors obtained using the ROR algorithm. (A) Infliximab had 10 positive signals. (B) Adalimumab had no positive signal. (C) Certolizumab pegol had two positive signals. (D) Golimumab had seven positive signals.

Across the four TNF-α inhibitors, we identified 36 Preferred Terms (PTs) associated with infective pneumonia-related adverse events, with infliximab having the highest number of PTs and golimumab the lowest. Infliximab had 10 positive signals, including tuberculosis, pulmonary tuberculosis, pneumocystis jirovecii pneumonia, histoplasmosis, pneumonia bacterial, pneumonia legionella, bronchopulmonary aspergillosis, tuberculous pleurisy, pneumonia cryptococcal, blastomycosis. Golimumab had seven positive signals, including pneumonia, tuberculosis, pulmonary tuberculosis, bronchopulmonary aspergillosis, pneumonia legionella, pneumonia bacterial, and COVID-19 pneumonia. Certolizumab pegol had only two positive signals: pneumonia and pneumonia klebsiella. However, adalimumab did not show signals of infective pneumonia. Table 3 displays the signal strength for Preferred Terms with ≥3 occurrences across the four drugs. We further conducted in-depth signal mining and analysis of infective pneumonia-associated Preferred Terms using the Reporting Odds Ratio (ROR) method. Fig 2 presents the forest plot of Preferred Terms for four TNF-α inhibitors obtained using the ROR algorithm. Moreover, the forest plot revealed that adverse events such as pneumonia, tuberculosis, and pulmonary tuberculosis occur with the highest frequency and exhibit strong signal values.

**Table 3:** The signal strength of infective pneumonia AEs related to TNF-α inhibitors.

		Infliximab(n = 3858)				Adalimumab(n = 2819)				Certolizumab pegol (n = 297)				Golimumab(n = 202)		
PT	Cases	ROR(95%Cl)	IC(IC025)	Signal	Cases	ROR(95%Cl)	IC(IC025)	Signal	Cases	ROR(95%Cl)	IC(IC025)	Signal	Cases	ROR(95%Cl)	IC(IC025)	Signal
Pneumonia	1942	0.92 (0.88 - 0.97)	−0.08 (−0.16)		2078	0.66 (0.63 - 0.7)	−0.4 (−0.47)		254	1.33 (1.18 - 1.51)	0.4 (0.21)	Y	151	2.82 (2.4 - 3.31)	1.46 (1.22)	Y
Tuberculosis	656	3.74 (3.32 - 4.22)	1.07 (0.93)	Y	242	0.46 (0.4 - 0.54)	−0.78 (−0.98)		17	0.61 (0.38 - 0.98)	−0.7 (−1.39)		16	1.94 (1.18 - 3.18)	0.94 (0.24)	Y
Pulmonary tuberculosis	327	6 (4.92 - 7.33)	1.31 (1.1)	Y	58	0.23 (0.18 - 0.31)	−1.6 (−2)						10	2.88 (1.54 - 5.4)	1.51 (0.63)	Y
Pneumocystis jirovecii pneumonia	160	2.19 (1.77 - 2.7)	0.71 (0.43)	Y	23	0.12 (0.08 - 0.18)	−2.5 (−3.11)						4	1.54 (0.57 - 4.13)	0.62 (−0.68)	
Histoplasmosis	143	3.42 (2.66 - 4.4)	1.02 (0.72)	Y	45	0.36 (0.26 - 0.5)	−1.06 (−1.52)									
Pneumonia bacterial	96	2.32 (1.76 - 3.06)	0.75 (0.4)	Y	30	0.29 (0.2 - 0.43)	−1.34 (−1.89)		4	0.76 (0.28 - 2.03)	−0.39 (−1.7)		8	5.45 (2.69 - 11.07)	2.4 (1.42)	Y
Pneumonia legionella	89	3.19 (2.34 - 4.37)	0.98 (0.6)	Y	40	0.55 (0.39 - 0.79)	−0.59 (−1.09)		3	0.7 (0.22 - 2.2)	−0.5 (−1.96)		6	5.13 (2.27 - 11.61)	2.31 (1.2)	Y
Covid-19 pneumonia	53	1.04 (0.76 - 1.43)	0.04 (−0.41)		24	0.25 (0.16 - 0.39)	−1.51 (−2.13)		5	1.01 (0.42 - 2.47)	0.02 (−1.18)		4	2.96 (1.1 - 7.97)	1.54 (0.23)	Y
Bronchopulmonary aspergillosis	44	2.05 (1.38 - 3.05)	0.66 (0.14)	Y	15	0.3 (0.17 - 0.51)	−1.31 (−2.08)						3	4.13 (1.31 - 13.04)	2.01 (0.54)	Y
Tuberculous pleurisy	32	5.36 (2.9 - 9.9)	1.26 (0.61)	Y	3	0.11 (0.03 - 0.36)	−2.57 (−4.07)									
Atypical pneumonia	31	0.86 (0.57 - 1.29)	−0.16 (−0.74)		50	1.14 (0.8 - 1.64)	0.11 (−0.37)		4	1.26 (0.47 - 3.43)	0.33 (−0.99)					
Lung abscess	31	1.34 (0.87 - 2.08)	0.29 (−0.3)		20	0.48 (0.29 - 0.78)	−0.75 (−1.45)		4	1.9 (0.7 - 5.21)	0.89 (−0.44)					
Pneumonia fungal	30	1.42 (0.91 - 2.23)	0.34 (−0.26)		37	1.32 (0.86 - 2.04)	0.24 (−0.33)									
Coccidioidomycosis	22	1.13 (0.68 - 1.87)	0.12 (−0.57)		21	0.69 (0.41 - 1.15)	−0.36 (−1.06)									
Pneumonia cryptococcal	20	2.64 (1.41 - 4.96)	0.85 (0.07)	Y	6	0.3 (0.13 - 0.71)	−1.3 (−2.48)									
Chlamydial infection	18	1.74 (0.95 - 3.17)	0.52 (−0.27)		4	0.16 (0.06 - 0.46)	−2.06 (−3.42)									
Pneumonia streptococcal	17	2.14 (1.12 - 4.08)	0.69 (−0.14)		8	0.45 (0.21 - 0.99)	−0.81 (−1.88)		3	3.25 (1 - 10.59)	1.61 (0.09)					
Acute pulmonary histoplasmosis	15	2.09 (1.06 - 4.15)	0.68 (−0.2)		6	0.36 (0.15 - 0.88)	−1.06 (−2.26)									
Pneumonia mycoplasmal	14	1.76 (0.89 - 3.48)	0.53 (−0.36)		8	0.51 (0.23 - 1.12)	−0.69 (−1.76)									
Pneumonia viral	13	0.59 (0.32 - 1.09)	−0.57 (−1.42)		24	0.9 (0.54 - 1.47)	−0.1 (−0.77)									
Pneumonia pseudomonal	12	2.15 (1 - 4.66)	0.7 (−0.28)		10	1.03 (0.47 - 2.26)	0.02 (−1.01)									
Pneumonia staphylococcal	11	1.2 (0.59 - 2.47)	0.18 (−0.78)		11	0.79 (0.38 - 1.61)	−0.23 (−1.19)									
Blastomycosis	10	6.28 (1.97 - 20.03)	1.33 (0.18)	Y												
Pulmonary sepsis	9	1.26 (0.56 - 2.8)	0.23 (−0.84)		8	0.69 (0.3 - 1.58)	−0.35 (−1.46)									
Pneumonia klebsiella	9	0.87 (0.41 - 1.86)	−0.15 (−1.18)		10	0.66 (0.32 - 1.37)	−0.41 (−1.4)		3	3.57 (1.09 - 11.7)	1.74 (0.21)	Y				
Post procedural pneumonia	7	0.8 (0.34 - 1.87)	−0.24 (−1.39)		13	1.33 (0.64 - 2.77)	0.24 (−0.69)									
Infectious pleural effusion	7	2.51 (0.88 - 7.16)	0.81 (−0.45)		5	0.91 (0.31 - 2.72)	−0.08 (−1.48)									
Pneumonia pneumococcal	7	1.26 (0.51 - 3.11)	0.23 (−0.96)		4	0.39 (0.13 - 1.15)	−0.99 (−2.42)									
Septic pulmonary embolism	7	4.4 (1.29 - 15.02)	1.16 (−0.16)													
Pneumonia cytomegaloviral	6	0.58 (0.24 - 1.41)	−0.6 (−1.8)													
Pneumonia influenzal	6	1.26 (0.47 - 3.35)	0.23 (−1.05)		6	0.82 (0.31 - 2.19)	−0.18 (−1.46)									
Pulmonary histoplasmosis	6	7.54 (1.52 - 37.35)	1.4 (−0.06)													
Haemophilus infection	5	1.79 (0.57 - 5.65)	0.55 (−0.87)		4	0.82 (0.25 - 2.73)	−0.18 (−1.7)									
Candida pneumonia	3	2.51 (0.51 - 12.45)	0.81 (−1)													
Pneumonia escherichia					3	1.64 (0.33 - 8.14)	0.4 (−1.41)									
Pneumonia necrotising					3	0.62 (0.16 - 2.32)	−0.47 (−2.14)									

## 4. Discussion

Tumor necrosis factor is crucial in mediating the immune response during both acute and chronic inflammation [15]. TNF antagonists, available as anti-TNF monoclonal antibodies or TNF fusion proteins, have become the most important treatment for inflammatory bowel disease [16]. Currently, the main TNF-α inhibitors used to treat inflammatory bowel disease are infliximab, adalimumab, certolizumab pegol, and golimumab. TNF-α inhibitors are a type of immunomodulator. They may trigger changes in immune responses, increasing susceptibility to infectious diseases. This susceptibility is particularly pronounced in diseases caused by common respiratory pathogens [17,18]. Among individuals receiving TNF-α inhibitors, mycobacterium tuberculosis accounts for 12.5–59% of all infections; pneumocystis jirovecii constitutes 20% of all non-viral infections; and the incidence of legionella pneumophila infections is 13–21 times higher compared to the general population [7]. In this study, we employed pharmacovigilance methods to thoroughly explore the complex relationship between TNF-α inhibitors and their associated infective pneumonia, ultimately assessing their post-marketing safety.

This large-scale pharmacovigilance study, analyzing 7,176 reports, revealed substantial heterogeneity in pneumonia risk profiles among different TNF-α inhibitors, with important implications for clinical decision-making and patient management strategies. Infliximab exhibited the broadest and most severe infection spectrum, associated with 10 different types of infective pneumonia, including tuberculosis, pneumocystis pneumonia, and various fungal pneumonias. This pattern aligns with infliximab’s mechanism as a chimeric monoclonal antibody that achieves complete and sustained TNF-α neutralization, profoundly disrupting multiple pulmonary defense mechanisms [19]. TNF-α plays a crucial role in granuloma formation and macrophage activation against intracellular pathogens, explaining the influential association with granulomatous infections [20]. TNF-α inhibitors disrupt granuloma formation by reducing pro-inflammatory signals, leading to the apoptosis of immune cells that maintain the granulomas, which allows trapped Mycobacterium tuberculosis to escape and cause active tuberculosis [21]. The broad spectrum of bacterial and fungal pneumonia associated with infliximab suggests it may more comprehensively impair pulmonary defenses than other agents in this class.

The risk profile of golimumab presents several noteworthy features. While showing fewer signals than infliximab, it maintained associations with tuberculosis and various bacterial pneumonias. Although golimumab is a human IgG1 monoclonal antibody, its binding affinity and neutralizing properties for TNF-α may differ from those of other TNF-α inhibitors [22]. The COVID-19 pneumonia signal reflects temporal reporting patterns during the pandemic and suggests that TNF antagonists may be associated with a higher risk of severe COVID-19 in patients with IBD [23]. Certolizumab pegol demonstrated only two infective pneumonia signals. Its unique structure as a PEGylated Fab’ fragment lacking an Fc region may explain this advantage, as it avoids Fc-mediated immune effects contributing to infection risk with other TNF-α inhibitors [24]. Nonetheless, less frequent use or underreporting may also influence the relatively low number of reports. Despite its widespread use, adalimumab exhibited no positive disproportionality signals for infective pneumonia in this analysis. While this could suggest a more favorable respiratory safety profile, caution must be exercised in interpreting this finding. The absence of signals does not equate to a lack of risk. Instead, it may be attributable to reporting variability, pharmacovigilance biases, or differences in patient demographics. Prior studies have indeed identified an elevated risk of infections with adalimumab, including pneumonia and reactivation of latent tuberculosis, particularly in combination with other immunosuppressants [25].

In our study, TNF-α inhibitors were closely associated with infective pneumonia. Our study found that the most common infections were those related to tuberculosis. Infection with mycobacterium tuberculosis can lead to a range of diseases, from an asymptomatic latent phase to severe pneumonia. If not treated promptly, this infection can potentially progress to a fatal condition. A nationwide population-based study in South Korea found that among IBD patients treated with anti-TNF-α therapy, the incidence rates of tuberculosis were significantly higher than that among all IBD patients [26]. Therefore, for patients suspected of having latent or active pulmonary tuberculosis, anti-TNF-α therapy should be deferred, and anti-tuberculosis treatment should be carried out according to national guidelines. In countries and regions with a high prevalence of tuberculosis, when diagnosing IBD, clinical examination should be intensified and combined with necessary diagnostic tests. Although guidelines generally recommend screening for tuberculosis infection before initiating TNF-α inhibitor therapy, TNF-α inhibitors can not only reactivate latent tuberculosis but also increase susceptibility to new infections. Therefore, it is crucial to emphasize the importance of thorough infection monitoring throughout treatment [27]. Additionally, in IBD patients receiving anti-TNF-α therapy, clinical monitoring protocols should be individualized, emphasizing close surveillance of respiratory symptoms during the initial treatment phase, especially within the first 6–12 months following treatment initiation [28]. Our study provided actionable insights for clinicians, researchers, and healthcare decision-makers to improve patient safety and treatment outcomes in managing IBD.

Although this study provided valuable insights into infective pneumonia following TNF-α inhibitor use in patients with inflammatory bowel disease, it was essential to acknowledge the limitations of the FAERS database itself. FAERS is a spontaneous reporting system with varying report quality, possibly leading to analytical biases. It is also difficult to control for confounding factors such as age, dosage, comorbidities, drug interactions, or other factors that may influence adverse events. Due to the possibility of incomplete reporting and reporting bias, our results must be interpreted cautiously. Additionally, the data in the FAERS database cannot directly establish causality, so we cannot solely rely on it to determine the causal relationship between TNF-α inhibitors and specific adverse events. Multiple factors, including drug characteristics, individual differences, and underlying conditions, influence the occurrence of adverse events. Future studies may consider adopting stricter prospective research methodologies that combine clinical trials with epidemiological investigations to gain a more comprehensive and precise understanding. This approach would enable a more accurate evaluation of the safety risks associated with TNF-α inhibitors.

## Conclusions

Our pharmacovigilance analysis of real-world data in the FEARS database revealed infective pneumonia associated with TNF-α inhibitors. Except for adalimumab, the other three TNF-α inhibitors showed positive signals related to infective pneumonia, with tuberculosis-related diseases being the most common infections. Infliximab was associated with the highest number of adverse events related to infective pneumonia, and it also had the most positive signals. Inflammatory bowel disease patients should be screened for tuberculosis infection before starting treatment with TNF-α inhibitors and thoroughly monitored for tuberculosis infection throughout treatment. However, further clinical research is still needed to validate these findings and gain a deeper understanding of the safety of tumor necrosis factor-α inhibitors.

## Supporting information

S1 Table PTs for all infective pneumonia events reported in the FAERS database. (DOCX)

S2 Table Two major algorithms used for signal detection. (DOCX)
