# Supplementary material for: Infective pneumonia following the use of tumor necrosis factor-α inhibitors in inflammatory bowel disease patients: A real-world disproportionality analysis of the FDA Adverse Event Reporting System (FAERS) database
Source: PLoS One. 2025 Aug 5;20(8):e0317242. doi: 10.1371/journal.pone.0317242 (PMC12324135; doi:10.1371/journal.pone.0317242)
Supplement: S1 Table — (DOCX) [file pone.0317242.s001.docx]

**Supplementary Table S1. PTs for all infective pneumonia events reported in the FAERS database.**

| NO. | Code | PT | SMQ | Scope |
| --- | --- | --- | --- | --- |
| 1 | 10000628 | Actinomycotic pulmonary infection | Infective pneumonia | Narrow |
| 2 | 10001027 | Acute pulmonary histoplasmosis | Infective pneumonia | Narrow |
| 3 | 10071075 | Atypical mycobacterial pneumonia | Infective pneumonia | Narrow |
| 4 | 10003757 | Atypical pneumonia | Infective pneumonia | Narrow |
| 5 | 10005098 | Blastomycosis | Infective pneumonia | Narrow |
| 6 | 10006473 | Bronchopulmonary aspergillosis | Infective pneumonia | Narrow |
| 7 | 10069657 | Burkholderia cepacia complex infection | Infective pneumonia | Narrow |
| 8 | 10069748 | Burkholderia pseudomallei infection | Infective pneumonia | Narrow |
| 9 | 10053158 | Candida pneumonia | Infective pneumonia | Narrow |
| 10 | 10061041 | Chlamydial infection | Infective pneumonia | Narrow |
| 11 | 10009115 | Chronic pulmonary histoplasmosis | Infective pneumonia | Narrow |
| 12 | 10009825 | Coccidioidomycosis | Infective pneumonia | Narrow |
| 13 | 10084381 | Coronavirus pneumonia | Infective pneumonia | Narrow |
| 14 | 10084380 | COVID-19 pneumonia | Infective pneumonia | Narrow |
| 15 | 10065680 | Embolic pneumonia | Infective pneumonia | Narrow |
| 16 | 10054218 | Enterobacter pneumonia | Infective pneumonia | Narrow |
| 17 | 10061190 | Haemophilus infection | Infective pneumonia | Narrow |
| 18 | 10077933 | Haemorrhagic pneumonia | Infective pneumonia | Narrow |
| 19 | 10019143 | Hantavirus pulmonary infection | Infective pneumonia | Narrow |
| 20 | 10065046 | Herpes simplex pneumonia | Infective pneumonia | Narrow |
| 21 | 10020141 | Histoplasmosis | Infective pneumonia | Narrow |
| 22 | 10071699 | Infectious pleural effusion | Infective pneumonia | Narrow |
| 23 | 10025028 | Lung abscess | Infective pneumonia | Narrow |
| 24 | 10085550 | Metapneumovirus pneumonia | Infective pneumonia | Narrow |
| 25 | 10055088 | Miliary pneumonia | Infective pneumonia | Narrow |
| 26 | 10080986 | Paracancerous pneumonia | Infective pneumonia | Narrow |
| 27 | 10078883 | Parasitic pneumonia | Infective pneumonia | Narrow |
| 28 | 10061351 | Pleural infection | Infective pneumonia | Narrow |
| 29 | 10067334 | Pleural infection bacterial | Infective pneumonia | Narrow |
| 30 | 10085959 | Pleurisy bacterial | Infective pneumonia | Narrow |
| 31 | 10052761 | Pleurisy viral | Infective pneumonia | Narrow |
| 32 | 10073755 | Pneumocystis jirovecii pneumonia | Infective pneumonia | Narrow |
| 33 | 10035664 | Pneumonia | Infective pneumonia | Narrow |
| 34 | 10079866 | Pneumonia acinetobacter | Infective pneumonia | Narrow |
| 35 | 10035665 | Pneumonia adenoviral | Infective pneumonia | Narrow |
| 36 | 10035667 | Pneumonia anthrax | Infective pneumonia | Narrow |
| 37 | 10060946 | Pneumonia bacterial | Infective pneumonia | Narrow |
| 38 | 10035672 | Pneumonia bordetella | Infective pneumonia | Narrow |
| 39 | 10035673 | Pneumonia chlamydial | Infective pneumonia | Narrow |
| 40 | 10067565 | Pneumonia cryptococcal | Infective pneumonia | Narrow |
| 41 | 10035676 | Pneumonia cytomegaloviral | Infective pneumonia | Narrow |
| 42 | 10035699 | Pneumonia escherichia | Infective pneumonia | Narrow |
| 43 | 10061354 | Pneumonia fungal | Infective pneumonia | Narrow |
| 44 | 10035702 | Pneumonia haemophilus | Infective pneumonia | Narrow |
| 45 | 10065246 | Pneumonia helminthic | Infective pneumonia | Narrow |
| 46 | 10035703 | Pneumonia herpes viral | Infective pneumonia | Narrow |
| 47 | 10035714 | Pneumonia influenzal | Infective pneumonia | Narrow |
| 48 | 10035717 | Pneumonia klebsiella | Infective pneumonia | Narrow |
| 49 | 10035718 | Pneumonia legionella | Infective pneumonia | Narrow |
| 50 | 10035722 | Pneumonia measles | Infective pneumonia | Narrow |
| 51 | 10035723 | Pneumonia moraxella | Infective pneumonia | Narrow |
| 52 | 10035724 | Pneumonia mycoplasmal | Infective pneumonia | Narrow |
| 53 | 10055672 | Pneumonia necrotising | Infective pneumonia | Narrow |
| 54 | 10035727 | Pneumonia parainfluenzae viral | Infective pneumonia | Narrow |
| 55 | 10035728 | Pneumonia pneumococcal | Infective pneumonia | Narrow |
| 56 | 10079867 | Pneumonia proteus | Infective pneumonia | Narrow |
| 57 | 10035731 | Pneumonia pseudomonal | Infective pneumonia | Narrow |
| 58 | 10035732 | Pneumonia respiratory syncytial viral | Infective pneumonia | Narrow |
| 59 | 10035733 | Pneumonia salmonella | Infective pneumonia | Narrow |
| 60 | 10079868 | Pneumonia serratia | Infective pneumonia | Narrow |
| 61 | 10035734 | Pneumonia staphylococcal | Infective pneumonia | Narrow |
| 62 | 10035735 | Pneumonia streptococcal | Infective pneumonia | Narrow |
| 63 | 10067566 | Pneumonia toxoplasmal | Infective pneumonia | Narrow |
| 64 | 10035736 | Pneumonia tularaemia | Infective pneumonia | Narrow |
| 65 | 10035737 | Pneumonia viral | Infective pneumonia | Narrow |
| 66 | 10053026 | Pneumonic plague | Infective pneumonia | Narrow |
| 67 | 10066590 | Post procedural pneumonia | Infective pneumonia | Narrow |
| 68 | 10086752 | Pulmonary blastomycosis | Infective pneumonia | Narrow |
| 69 | 10037374 | Pulmonary echinococciasis | Infective pneumonia | Narrow |
| 70 | 10086750 | Pulmonary histoplasmosis | Infective pneumonia | Narrow |
| 71 | 10078354 | Pulmonary mucormycosis | Infective pneumonia | Narrow |
| 72 | 10080435 | Pulmonary nocardiosis | Infective pneumonia | Narrow |
| 73 | 10080468 | Pulmonary paracoccidioidomycosis | Infective pneumonia | Narrow |
| 74 | 10051739 | Pulmonary sepsis | Infective pneumonia | Narrow |
| 75 | 10080480 | Pulmonary sporotrichosis | Infective pneumonia | Narrow |
| 76 | 10037434 | Pulmonary syphilis | Infective pneumonia | Narrow |
| 77 | 10068184 | Pulmonary trichosporonosis | Infective pneumonia | Narrow |
| 78 | 10037440 | Pulmonary tuberculosis | Infective pneumonia | Narrow |
| 79 | 10057102 | Pyopneumothorax | Infective pneumonia | Narrow |
| 80 | 10083093 | Septic pulmonary embolism | Infective pneumonia | Narrow |
| 81 | 10044755 | Tuberculosis | Infective pneumonia | Narrow |
| 82 | 10045104 | Tuberculous pleurisy | Infective pneumonia | Narrow |
| 83 | 10074254 | Varicella zoster pneumonia | Infective pneumonia | Narrow |
