# Supplementary material for: Infective pneumonia following the use of tumor necrosis factor-α inhibitors in inflammatory bowel disease patients: A real-world disproportionality analysis of the FDA Adverse Event Reporting System (FAERS) database
Source: PLoS One. 2025 Aug 5;20(8):e0317242. doi: 10.1371/journal.pone.0317242 (PMC12324135; doi:10.1371/journal.pone.0317242)
Supplement: S2 Table — (DOCX) [file pone.0317242.s002.docx]

**Supplementary Table S2. Two major algorithms used for signal detection.**

| Algorithms | Equation | Criteria |
| --- | --- | --- |
| ROR | ROR=ad/b/c | lower limit of 95% CI>1, N≥3 |
|  | 95%CI=e^ln(ROR)±1.96(1/a+1/b+1/c+1/d)^0.5^ |  |
| BCPNN | IC=log_2_a(a+b+c+d)(a+c)(a+b) | IC025>0 |
|  | 95%CI= E(IC) ± 2V(IC)^0.5 |  |

Equation: a, number of reports containing both the target drug and target adverse drug reaction; b, number of reports containing other adverse drug reaction of the target drug; c, number of reports containing the target adverse drug reaction of other drugs; d, number of reports containing other drugs and other adverse drug reactions. 95%CI, 95% confidence interval; *N*, the number of reports; IC, information component; IC025, the lower limit of 95% CI of the IC; E(IC), the IC expectations; V(IC), the variance of IC.
